# The Effect of a Digital Manufacturing Technique, Preparation Taper, and Finish Line Design on the Marginal Fit of Temporary Molar Crowns: An In-Vitro Study

**DOI:** 10.3390/biomedicines11020570

**Published:** 2023-02-15

**Authors:** Maryam H. Mugri, Harisha Dewan, Mohammed E. Sayed, Fawzia Ibraheem Shaabi, Hanan Ibrahim Hakami, Hossam F. Jokhadar, Nasser M. Alqahtani, Ahid Amer Alshahrani, Abdullah S. Alabdullah, Abdullah Hasan Alshehri, Mohammed Hussain Dafer Al Wadei, Fatimah Yahya Arif, Ebtihag H. Adawi, Bandar M. A. Al-Makramani, Hitesh Chohan

**Affiliations:** 1 Department of Maxillofacial Surgery and Diagnostic Sciences, College of Dentistry, Jazan University, Jazan 45142, Saudi Arabia; 2 Department of Prosthetic Dental Sciences, College of Dentistry, Jazan University, Jazan 45142, Saudi Arabia; 3 Primary Care Administration, Ministry of Health, Jazan 45142, Saudi Arabia; 4 Department of Oral and Maxillofacial Prosthodontics, Faculty of Dentistry, King Abdulaziz University, Jeddah 21589, Saudi Arabia; 5 Department of Prosthetic Dental Sciences, College of Dentistry, King Khalid University, Abha 61413, Saudi Arabia; 6 Department of Dental Technology, Applied Medical Sciences College, King Khalid University, Abha 61413, Saudi Arabia; 7 King Faisal Medical City, Ministry of Health, Abha 62586, Saudi Arabia; 8 Department of Restorative Dental Sciences, College of Dentistry, King Khalid University, Abha 61413, Saudi Arabia; 9 Private Practice, Jazan University, Jazan 45142, Saudi Arabia; 10 Department of Restorative Dental Sciences, College of Dentistry, Jazan University, Jazan 45142, Saudi Arabia

**Keywords:** temporary crowns, CAD/CAM milled, 3D printed, preparation taper, finish line, marginal gaps

## Abstract

The aim of this study is to investigate the combined effect of a digital manufacturing technique (subtractive vs. additive), preparation taper (10° vs. 20° TOC), and finish line (chamfer vs. shoulder) on the marginal adaptation of temporary crowns following cementation with a compatible temporary cement. Four mandibular first molar typodont teeth were prepared for full coverage crowns with standard 4 mm preparation height as follows: 10° TOC with the chamfer finish line, 10° TOC with the shoulder finish line, 20° TOC with the chamfer finish line and 20° TOC with the shoulder finish line. Each of the four preparation designs were subdivided into two subgroups to receive CAD/CAM milled and 3D-printed crowns (*n* = 10). A total of 80 temporary crowns (40 CAD/CAM milled and 40 3D-printed) were cemented to their respective die using clear temporary recement in the standard cementation technique. The samples were examined under a stereomicroscope at ×100 magnification following calibration. Linear measurements were performed at seven equidistant points on each axial surface and five equidistant points on each proximal surface. One-way ANOVA analysis and Tukey HSD (Honestly Significance Difference) were performed. The best marginal fit was seen in group 8, while the poorest fit was noted in group 2. Shoulder finish lines and 10° TOC resulted in higher marginal gaps, especially in CAD/CAM milled group. The selection of 3D-printed crowns may provide a better marginal fit within the range of clinical acceptability. Marginal gaps were within clinical acceptability (50 and 120 µm) in all groups except group 2.

## 1. Introduction

A temporary crown is defined as a restoration that is fabricated to maintain or improve esthetics, stabilization, or function for a finite period of time and replaced afterward by a definitive crown [1]. Temporary crowns and prostheses are one of the most common dental treatments in dentate and partially dentate patients [2]. A nationwide survey indicated that one-third of dentate adults in the United Kingdom have received a minimum of one tooth crown, one-fifth have received 1–2 crowns, and around 5% presented with two or more crowns, while 40 million crowns were delivered each year clinically in the United States alone [3,4].

To maintain periodontium health and to achieve a good survival rate of a prosthesis, in terms of function and esthetics, importance must be given to certain biological considerations. Although several criteria should be present in an ideal temporary crown, the marginal fit is the most important criterion for the biological protection of the prepared tooth [5]. Intimate adaptation of the temporary crown during the provisional phase of restorative treatment helps to achieve proper gingival contour for cleansability [6] and eliminate microleakage, therefore lowering the risk of pulpal irritation and recurrent caries in restored teeth [7]. Additionally, while restoring missing space with implants, the well-fitted provisional prosthesis is used to develop proper contours after the soft tissue around the implants has achieved good volume.

Several factors have been identified in the literature to affect the fit of the crown. These factors could be related to tooth preparation, such as preparation height [8], total occlusal convergence [8,9,10], design of finish line [11,12,13,14], and surface topography [15], or related to the type and fabrication technique of the crown itself [16,17,18]. Luting cement type [19], cementation technique [20], and availability of cement spacer [21] are additional factors that affect the fit of crown restorations.

These factors were evaluated exclusively in definitive crown restorations. The current literature is lacking with regard to the effect of these factors on the fit of temporary crown restorations. However, certain clinical situations require a prolonged temporization phase [22,23] prior to initiating the phase of the definitive restorations.

The use of computer-aided designing and computer-aided manufacturing (CAD/CAM)) has proven to be a boon in the management of dental cases. These procedures use pre-polymerized resin blocks to obtain the desired shape of the provisional prosthesis [24]. Conventional provisional resins have been compared to CAD/CAM milled provisional resins in terms of physical and mechanical properties, and the latter has been proven to be superior [25]. Additive manufacturing/three-dimensional (3D) printing techniques have become popular in recent times, in which the desired shape is achieved by using a layering technique. Various methods of the 3D printing technique are Digital light processing (DLP), Selective Laser Sintering (SLS), and Stereolithography (SLA). The main advantage of the 3D printing technique is the decrease in wastage of raw materials and reduction in manufacturing time. There are varied results in previous studies comparing the properties of conventional provisional 3D-printed resins (used for fabricating provisional crowns and FDPs) and those of CAD/CAM-milled provisional resins [25].

Therefore, the aim of this study is to evaluate the effect of digital fabrication technique (milled and 3D printed), preparation taper (10 and 20 degrees of total occlusal convergence (TOC)) and finish line (chamfer and shoulder) on the marginal fit of temporary crowns that were cemented on standardized dies with one type of temporary cement. The null hypothesis can be framed that no statistically significant differences exist in marginal adaptation between milled and 3D printed temporary crowns cemented on prepared teeth models with variable degrees of TOC and finish lines.

## 2. Materials and Methods

The study protocol was approved in the Scientific Research Unit, College of Dentistry, Jazan University, on 31 May 2022 (Reference No. CODJU-2206F).

### 2.1. Materials

**Figure 1:**
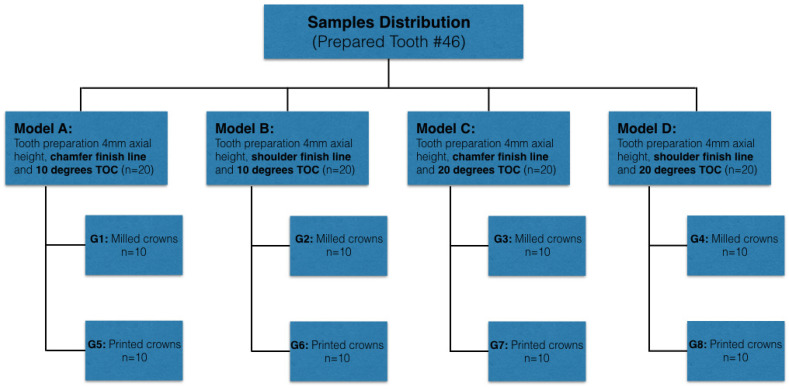
The study design used in the present study.

The materials, their composition, and the machines included in the present study are listed in Table 1. The study design is shown in Figure 1.

### 2.2. Preparation of the Master Models

A customized dental surveyor was used to fix a high-speed handpiece to perform standardized teeth preparations (mandibular left molar) on the typodont models (Frasaco An-4 Puk, Pok) [26]. The study setup was performed in the phantom laboratory of the College, in the same location for all the preparations. The occlusal plane was kept parallel to the horizon, and standard models were used. Guidelines for the preparation are listed in Table 2. All the sharp points or line angles were rounded off. To determine the molar tooth preparation angle to its long axis, a digital protractor (ATRIUM 200 mm Digital Electronic Angle Finder Goniometer Measuring Ruler Atrium digital protractor) was used with an accuracy of ±0.1°. The use of putty indices on the models helped in placing the digital protractor repeatedly against the buccal surfaces for all models [27]. Thus, four preparations were completed as per the guidelines mentioned in Table 2.

The four preparations were converted into appropriate master models (Model A–D) by mounting the typodont teeth on poly (methyl methacrylate) resin (Quick resin, Ivoclar, Schaan, Liechtenstein) block. The design of the resin block was made compatible with the configuration of the testing machine holding clamp.

### 2.3. Preparation of Working Models

**Figure 2:**
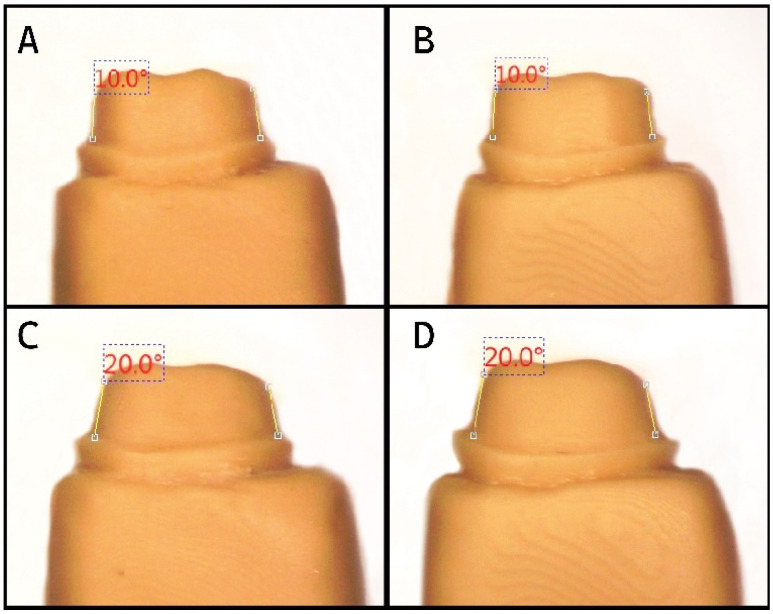
Using the stereomicroscope to verify taper (10° and 20° occlusal convergence) of the initial working models made with 3D-printed resin dies for Model (**A**–**D**).

A Bench Top Scanner (3 Shape, Copenhagen, Denmark, Model No. 4) was used to scan the four master models (Model A–D), and the design software was used to retrieve the scanned data. The 3D-printing machine (Asiga 3D printer, Alexandria, Australia, Serial Number: 70B3D5362C6A, Model Number: PN01233) and corresponding resin material (DentaModel, Asiga, Alexandria, Australia, Lot: MO/16020) were used to print the four working models. To verify the taper before printing the rest of the working models, a personal computer with a stereomicroscope connected with a USB CCD camera (Amscope, Irvine, CA, USA) and compatible measurement software (Version No. 3.7.12924) was used (Figure 2). For the final working models, 80 (20 for each master model) were 3D-printed once the taper was verified.

### 2.4. Preparation of the Coping Specimens

The prepared working models were used to fabricate provisional copings either by 3D-milling or 3D-printing. The 8 groups are listed in Table 3.

Separate STL files were generated and later processed after scanning the 80 working models. The coping was designed with an incisal ring of a 4 mm external diameter and a 2 mm internal diameter, and a cement space of 50 μm was set (Figure 3.1). A five-axis milling machine (DG SHAPE, Roland DGA, Irvine, CA, USA, Model: DWX-52D) and PMMA temporary crowns blocks (CopraTemp Shade A1, WhitePeaks Dental Solutions GmbH, Wesel, Germany) were used to produce 40 samples of CAD/CAM milling copings (Groups 1, 2, 3, and 4) from the saved STL files (Figure 3.2). The same printing system and the corresponding resin material (DentaTooth Shade A1, Asiga, Alexandria, Australia, Lot: MO/08782) was used to produce 40 samples of 3D-printed copings (Groups 5, 6, 7, and 8) on the remaining working models (Figure 3.3). All the copings were tried on their working models to verify the fit.

### 2.5. Cementation of the Copings

Kerr Temp-Bond Clear cement (Kerr, Romulus, MI, USA) was used to cement the crowns according to the manufacturer’s instructions. Crowns were placed on the working models, and 50 N force was applied for cementation. After the initial set was obtained, excess cement was removed with the help of an explorer.

### 2.6. Measurement of Marginal Fit

**Figure 4:**
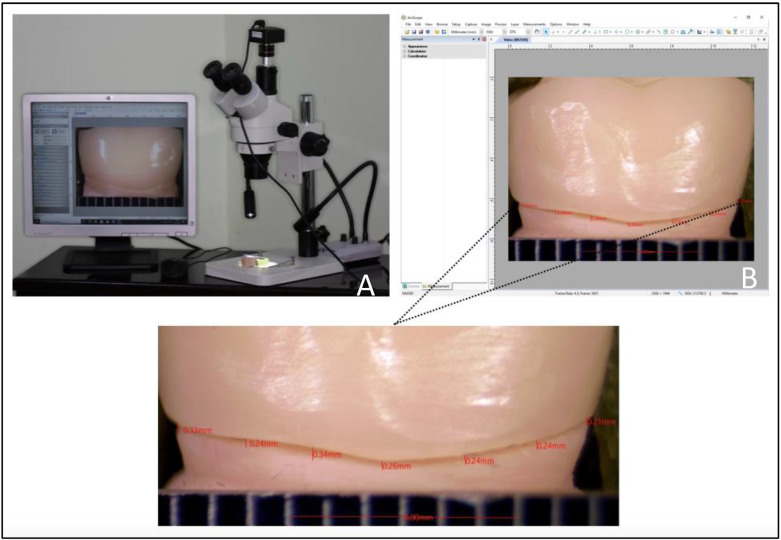
(**A**) Measurement setting with a stereomicroscope and mounted camera connected to a PC with compatible measurement software. (**B**) Seven-point measurements at equidistant points on the buccal surface on one of the study samples.

Following cementation, a putty placement index was made on the horizontal platform of the stereomicroscope to allow for standardized positioning of the samples upon measurements. The prototyped dies were made with offset flat cuts on axial and proximal surfaces at the level of the finish line to allow for the placement of a metallic ruler for precise calibration. Linear calibration was performed at a magnification of ×100. Seven measurements were performed at each axial surface (buccal and lingual) at equidistant points while on the proximal surfaces (mesial and distal) five equidistant points were used for gap measurements. A LED double gooseneck illuminator was used as a light source for accurate visualization (VMLIHL-20, Vision Scientific, Westland, MI, USA). Linear measurements of the gaps between the crown margin and finish line were performed using a compatible measurement software (Amscope Version No. 3.7.12924, Irvine, CA, USA) to evaluate marginal fit at all four surfaces (in mm) (Figure 4). All acquired data were entered into an Excel spreadsheet according to the sample distribution among the eight tested groups.

### 2.7. Statistical Analysis

A descriptive statistic was performed to calculate the mean and standard deviation. Statistical differences among the eight groups were made using one-way analysis of variance, ANOVA, and Tukey’s HSD posthoc tests. Data processing was performed using SPSS statistical software, version 20 (SPSS Inc., Chicago, IL, USA). The level of significance was set at *p* < 0.05 for all tests.

## 3. Results

**Figure 5:**
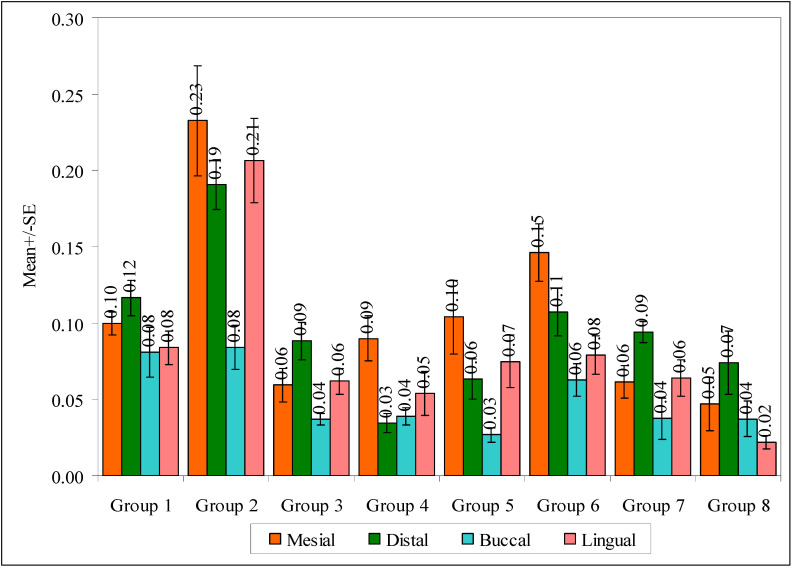
Comparison of eight groups with mean marginal gaps in different surfaces.

In Table 4, a significant difference was observed between the eight groups with mean marginal gap scores at the mesial surface (F = 9.5989, *p* = 0.0001), lingual surface (F = 13.2422, *p* = 0.0001), and total surface (F = 19.6429, *p* = 0.0001). It means that the mean marginal gap scores are different in the eight groups. At these surfaces, the mean marginal gap scores were highest in group 2 and lowest in group 8. The marginal gaps at the distal surface (F = 11.5135, *p* = 0.0001) and buccal surface (F = 3.9719, *p* = 0.0010) were also significantly different between the eight groups, in which total surfaces showed mean marginal gap scores as the highest in group 2 and lowest in group 8. A comparison of eight groups with mean marginal gaps in different regions is shown in Figure 5.

Further, to know the pairwise comparisons of eight groups with mean marginal gap scores, proximal marginal gap scores, and axial marginal gap scores, Tukey’s multiple posthoc test was applied. The results are presented in Table 5, Supplementary Tables S1 and S2, respectively.

**Figure 6:**
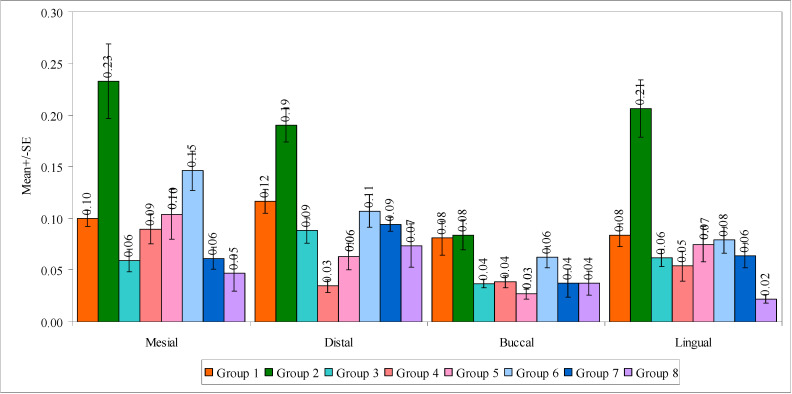
Comparison of four surfaces with mean marginal gap scores in the eight groups.

In a comparison of four regions with mean marginal gap scores in the eight groups by one-way ANOVA, no significant difference was observed between the four surfaces with mean marginal gap scores in group 1 (F = 1.8457, *p* = 0.1584). It means that the mean marginal gap scores were similar in four different surfaces in group 1. On the contrary, a significant difference was observed between four regions with mean marginal gap scores in group 2 (F = 6.6890, *p* = 0.0011), group 4 (F = 5.1191, *p* = 0.0047), group 5 (F = 3.8137, *p* = 0.0180) and group 6 (F = 6.1135, *p* = 0.0018). It means that the mean marginal gap scores are different on four different surfaces in these groups. In other words, the mean marginal gap scores were highest at the mesial surface and lowest at the buccal surface. Groups 3 (F = 5.0394, *p* = 0.0051) and 7 (F = 4.5462, *p* = 0.0084) showed a significant difference between the four surfaces with mean marginal gap scores, in which the mean marginal gap scores were highest at the distal surface and lowest at the buccal surfaces in both groups. No significant difference was observed between the four surfaces with the mean marginal gap scores in group 8 (F = 2.1245, *p* = 0.1142). It means that the mean marginal gap scores were similar in four different regions in group 8. A comparison of the four surfaces with mean marginal gap scores in the eight groups is shown in Figure 6.

Further, to know the pair wise comparisons of the four surfaces with mean marginal gap scores, the Tukey’s multiple post-hoc procedures were applied, and the results are presented in the above Table 6.

## 4. Discussion

The aim of this study is to evaluate the effect of digital fabrication technique (milled and 3D printed), preparation taper, and finish line on the marginal fit of temporary crowns.

Temporary and permanent crowns are manufactured by various methods. Subtractive manufacturing involves the milling of a solid block into a prosthesis. Though this technology has several advantages, such as better quality, reduced labor, and cost-effectiveness, there are certain disadvantages, such as difficulty in the fabrication of complex prostheses and wastage of material. Burs used for milling are also subjected to abrasive wear. Modern additive manufacturing or 3D printing techniques are now used by many dentists worldwide as it permits the manufacture of extremely complex shapes, reduces the working time, and diminishes the material wastage problem [28,29,30].

CAD/CAM fabricated provisional crowns are made up of resin blocks or PMMA [24,31]. The lack of polymerization shrinkage and heat generation are the two major advantages of these materials [32]. Whether the technician uses conventional methods or CAD/CAM, temporary crowns should have adequate fracture strength and adequate marginal integrity for clinical success [33]. A recent systematic review and meta-analysis has concluded that 3D-printed temporary crowns and FPD have presented superior marginal adaptation and internal fit when compared with the CAD/CAM milled and conventional counterparts [25]. To the best of the authors’ knowledge, the interactive effect of digital manufacturing technique (subtractive vs. additive), preparation taper (10° vs. 20° TOC), and finish line (chamfer vs. shoulder) on marginal fit of temporary crowns have not been investigated in the current literature.

Dental preparations use different finish lines [34]. Some studies investigated the provisional crowns for their marginal range measurement [35,36] and fracture strength [37]. The effect of chamfer and shoulder finish lines on the marginal integrity of temporary crowns was evaluated by Keyf et al. (1994) and found no statistically significant difference in marginal integrity between chamfer and shoulder finish lines [38].

2° to 6° of taper is suggested in the preparations by previous studies as it offers good retention and resistance [39]. This convergence angle is difficult to achieve in clinics. However, this is not applicable in clinical situations. A taper slightly more than this can compensate for any imperfections that may occur during the fabrication process, may give good seating space during cementation, and also facilitate the path of insertion. Too much taper may decrease retention and also cement failure [40]. The effect of varying taper on marginal integrity was not studied previously on the temporary prosthesis.

A crucial factor for the long-term survival of a prosthesis is the marginal fit [41,42,43,44,45]. Although there is a scarcity of scientific evidence literature, and the ideal would be to obtain closure with a gap of less than 25 µm, the clinically acceptable marginal gaps are considered to be between 50 and 120 µm [46,47]. A marginal discrepancy between 15 and 120 µm has been reported for milled zirconia restorations in several past studies [48,49,50,51,52]. A recent systematic review reported a marginal gap between 7.6 µm and 206.3 µm for milled monolithic crowns [49]. Although 3D printing of zirconia is gaining popularity, very few studies have been reported, especially those evaluating the marginal gaps [53,54,55].

Though the literature has researched comparing the marginal integrity of permanent restorations and the effect of the finish line and the convergence angle, their effects on the provisional crowns were not studied.

A significant difference was observed between eight groups with mean marginal gap scores of all surfaces (total) (F = 19.6429, *p* = 0.0001). It means that the mean marginal gap scores are different in eight different groups. In other words, the mean marginal gap scores are highest in group 2 and lowest in Group 8. Therefore, the null hypothesis that no statistically significant differences exist in marginal adaptation between milled and 3D printed temporary crowns cemented on prepared teeth models with variable degrees of TOC and finish lines is rejected. Additionally, the total mean marginal gap 45 microns (0.450 mm) is found for group 8, which was within the clinically acceptable range [46,47].

Most of the previous studies did not show significant differences in marginal adaption amongst the four surfaces except for one study [56], which stated that the facial and lingual margins exhibited significantly larger marginal discrepancies than the mesial and distal margins (explained by the greater amount of firing shrinkage at the facial and lingual surfaces). In our present study, a significant difference was observed between four regions with mean marginal gap scores in group 2 (F = 6.6890, *p* = 0.0011), group 4 (F = 5.1191, *p* = 0.0047), group 5 (F = 3.8137, *p* = 0.0180) and group 6 (F = 6.1135, *p* = 0.0018). The mean marginal gap scores were highest at the mesial surface and lowest at the buccal surface, which was contrary to the result obtained by Sulaiman et al. [56].

The outcome of this study provided clear guidelines that 3D-printed temporary crowns have better marginal fit than CAD/CAM milled temporary crowns, especially when a clinical scenario presented a combination of 20° TOC and shoulder finish line. Poor marginal fit for CAD/CAM milled temporary crowns was found in preparations with 10° TOC and shoulder finish line; in such a scenario, the digital design might be modified to provide a thicker cement spacer to allow better adaption and such crowns may require repair or reline with a resin material at chairside prior to cementation. As an alternative to that, 3D-printed temporary crowns can be considered an accurate alternative because they show a better marginal fit in this scenario.

## 5. Limitations

The type of tooth preparation used in this study may not reflect the actual clinical situations. For standardization purposes, tooth preparation dies were prototyped and multiplied using 3D-printing technologies to minimize variations among the study groups, which could be extremely difficult to achieve in case natural teeth were considered for sample preparation. Despite that, the mechanical properties of natural dentin and 3D-printed resin are close to each other. The temporary cement used in the study is clear to allow for better visualization of marginal gaps under the stereomicroscope.

In order to reinforce the results of the present study, further in vitro studies with increased sample sizes should be conducted. In vivo findings using randomised clinical trials would help in validating the clinical use of 3D-printed temporary crowns. The storage difficulties for the 3D printing slurry, expensive equipment, and fewer colour options available are the main challenges to the effective application of this technique in clinical dentistry. Additionally, very few studies have addressed the ability of 3D-printed zirconia to withstand the prosthetic load in the long term. Further research is essential for the clinical application of a 3D-printing technique in routine dental practice. Nonetheless, the present study has shown that the 3D-printing technique has great potential to be used for the fabrication of long-term provisional crowns.

## 6. Conclusions

Within the limitations of this study, the following conclusions can be drawn:

1. The best marginal fit was seen in group 8, while the poorest fit was noted in group 2.
2. Shoulder finish lines and 10° TOC resulted in higher marginal gaps, especially in CAD/CAM milled group. The selection of 3D-printed crowns may provide a better marginal fit within the range of clinical acceptability.
3. With the exception of one group (G2), the marginal gaps were within clinical acceptability (50 and 120 µm). Furthermore, 3D-printed crowns showed marginal gaps near the lower end of the clinical acceptability range, while marginal gaps of CAD/CAM milled crowns were near the upper end of that range.
4. CAD/CAM milled crowns and 3D printed crowns may be comparable to each other in preparations of 20° TOC, chamfer, and shoulder finish lines.

## Figures and Tables

**Figure 3:**
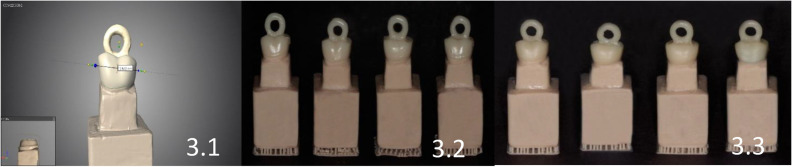
(**3.1**) STL file saved with incisal ring of a 4 mm external diameter and a 2 mm internal diameter and the cement space of 50 μm. (**3.2**) 3D-Milled crowns based on the generated file by the software from Model A–D. (**3.3**) 3D-Printed crowns based on the generated file by the software from Model A–D.

**Table 1:** The materials and machines included in the present study.

Materials/Machines	Manufacturer	Lot No.	Model No.	Composition
ATRIUM 200 mm Digital Electronic Angle Finder Goniometer Measuring Ruler Atrium digital protractor	Atrium Enterprise, Cardiff, UK		CR 2032	
Five-axis milling machine	DG SHAPE, Roland DGA, Irvine, CA, USA		DWX-52D	
3D-printing material	Asiga Pty Ltd., Alexandria, Australia	MO/16020		7,7,9(or 7,9,9)- trimethyl-4,13-dioxo-3,14-dioxa-5,12- diazahexadecane-1,16- diyl bismethacrylate, Tetrahydrofurfuryl methacrylate, Diphenyl(2,4,6- trimethylbenzoyl) phosphine oxide
3D-printing machine	Asiga 3D printer, Alexandria, Australia, Serial Number: 70B3D5362C6A		PN01233	
Copra temp	CopraTemp Shade A1, WhitePeaks Dental Solutions GmbH, Wesel, Germany	P10690		PMMA (polymethylmethacrylate)/pigments
Temp Bond Clear	Kerr, Romulus, MI, USA	8375246		Dibutyl phthalate, Hydroxyethylmethacrylate, Fumed silica, N-(2-Pyridyl)thiourea, Ethyldimethylaminobenzoate, Triclosan

**Table 2:** Guidelines for the preparation.

Model Number	Axial Height	Occlusal Convergence	Finish Line
A	4 mm	10°	Chamfer
B	4 mm	10°	Shoulder
C	4 mm	20°	Chamfer
D	4 mm	20°	Shoulder

**Table 3:** Eight groups of provisional copings fabricated either by 3D-milling or 3D-printing.

Groups	Description
GROUP 1	3D-Milled crowns (10 samples) based on the generated file by the software from Model A.
GROUP 2	3D-Milled crowns (10 samples) based on the generated file by the software from Model B.
GROUP 3	3D-Milled crowns (10 samples) based on the generated file by the software from Model C.
GROUP 4	3D-Milled crowns (10 samples) based on the generated file by the software from Model D.
GROUP 5	3D-Printed crowns (10 samples) based on the generated file by the software from Model A.
GROUP 6	3D-Printed crowns (10 samples) based on the generated file by the software from Model B.
GROUP 7	3D-Printed crowns (10 samples) based on the generated file by the software from Model C.
GROUP 8	3D-Printed crowns (10 samples) based on the generated file by the software from Model D.

**Table 4:** Comparison of eight groups with mean marginal gaps (in mm) in different regions by one way ANOVA.

Groups	Mesial		Distal		Buccal		Lingual		Total	
	Mean	SD	Mean	SD	Mean	SD	Mean	SD	Mean	SD
Group 1	0.0998	0.0243	0.1166	0.0365	0.0810	0.0518	0.0839	0.0352	0.0953	0.0273
Group 2	0.2328	0.1140	0.1906	0.0514	0.0841	0.0466	0.2063	0.0874	0.1785	0.0474
Group 3	0.0594	0.0341	0.0884	0.0385	0.0370	0.0126	0.0619	0.0267	0.0617	0.0154
Group 4	0.0900	0.0460	0.0346	0.0193	0.0389	0.0186	0.0540	0.0460	0.0544	0.0257
Group 5	0.1040	0.0762	0.0632	0.0416	0.0269	0.0149	0.0749	0.0541	0.0672	0.0378
Group 6	0.1462	0.0604	0.1074	0.0495	0.0626	0.0336	0.0791	0.0392	0.0988	0.0265
Group 7	0.0616	0.0332	0.0944	0.0220	0.0374	0.0421	0.0640	0.0379	0.0644	0.0195
Group 8	0.0470	0.0554	0.0738	0.0656	0.0373	0.0372	0.0220	0.0132	0.0450	0.0326
F-value	9.5989		11.5135		3.9719		13.2422		19.6429	
*p*-value	0.0001 *		0.0001 *		0.0010 *		0.0001 *		0.0001 *	

* *p* < 0.05 indicates a significant difference between the two groups; SD: Standard Deviation

**Table 5:** Pairwise comparison of the total mean marginal gaps between eight groups by Tukey’s multiple posthoc procedures.

Group	1	2	3	4	5	6	7	8
	MD/*p*-Value	MD/*p*-Value	MD/*p*-Value	MD/*p*-Value	MD/*p*-Value	MD/*p*-Value	MD/*p*-Value	MD/*p*-Value
1	X	−0.08/0.0001 *	0.03/0.2281	0.04/0.0689	0.03/0.4528	−0.00/1.0000	0.03/0.3264	0.05/0.0101 *
2	0.08/0.0001 *	X	0.12/0.0001 *	0.12/0.0001 *	0.11/0.0001 *	0.08/0.0001 *	0.11/0.0001 *	0.13/0.0001 *
3	−0.03/0.2281	−0.12/0.0001 *	X	0.01/0.9995	−0.01/0.9999	−0.04/0.1332	−0.00/1.0000	0.012/0.9239
4	−0.04/0.0689	−0.12/0.0001 *	−0.01/0.9995	X	−0.01/0.9808	−0.04/0.0350 *	−0.01/0.9958	0.01/0.9972
5	−0.03/0.4528	−0.11/0.0001 *	0.01/0.9999	0.01/0.9808	X	−0.03/0.3009	0.01/1.0000	0.02/0.7334
6	0.00/1.0000	−0.08/0.0001 *	0.04/0.1332	0.04/0.0350 *	0.03/0.3009	X	0.01/0.2026	0.06/0.0045 *
7	−0.03/0.3264	−0.11/0.0001 *	0.00/1.0000	0.01/0.9958	−0.01/1.0000	−0.01/0.2026	X	0.02/0.8472
8	−0.05/0.0101 *	−0.13/0.0001 *	−0.012/0.9239	−0.01/0.9972	−0.02/0.7334	−0.06/0.0045 *	−0.02/0.8472	X

*p < 0.05 indicates a significant difference between the two groups; MD: mean difference (difference in means); X: Not Applicable.

**Table 6:** Comparison of four surfaces with mean marginal gaps in eight groups by one way ANOVA.

Region	Group 1		Group 2		Group 3		Group 4		Group 5		Group 6		Group 7		Group 8	
	Mean	SD	Mean	SD	Mean	SD	Mean	SD	Mean	SD	Mean	SD	Mean	SD	Mean	SD
Mesial	0.10	0.02	0.23	0.11	0.06	0.03	0.09	0.05	0.10	0.08	0.15	0.06	0.06	0.03	0.05	0.06
Distal	0.12	0.04	0.19	0.05	0.09	0.04	0.03	0.02	0.06	0.04	0.11	0.05	0.09	0.02	0.07	0.07
Buccal	0.08	0.05	0.08	0.05	0.04	0.01	0.04	0.02	0.03	0.01	0.06	0.03	0.04	0.04	0.04	0.04
Lingual	0.08	0.04	0.21	0.09	0.06	0.03	0.05	0.05	0.07	0.05	0.08	0.04	0.06	0.04	0.02	0.01
F-value	1.8457		6.6890		5.0394		5.1191		3.8137		6.1135		4.5462		2.1245	
*p*-value	0.1564		0.0011 *		0.0051 *		0.0047 *		0.0180 *		0.0018 *		0.0084 *		0.1142	
Pair wise comparisons by Tukey’s multiple posthoc procedures																
Mesial vs. Distal	*p* = 0.7603		*p* = 0.6414		*p* = 0.1461		*p* = 0.0063 *		*p* = 0.3065		*p* = 0.2661		*p* = 0.1669		*p* = 0.5884	
Mesial vs. Buccal	*p* = 0.6922		*p* = 0.0011 *		*p* = 0.3439		*p* = 0.0129 *		*p* = 0.0103 *		*p* = 0.0018 *		*p* = 0.4133		*p* = 0.9673	
Mesial vs. Lingual	*p* = 0.7878		*p* = 0.8790		*p* = 0.9978		*p* = 0.1199		*p* = 0.5933		*p* = 0.0145 *		*p* = 0.9987		*p* = 0.6411	
Distal vs. Buccal	*p* = 0.1784		*p* = 0.0251 *		*p* = 0.0024 *		*p* = 0.9930		*p* = 0.4069		*p* = 0.1598		*p* = 0.0042*		*p* = 0.3243	
Distal vs. Lingual	*p* = 0.2400		*p* = 0.9712		*p* = 0.2061		*p* = 0.6105		*p* = 0.9576		*p* = 0.5383		*p* = 0.2209		*p* = 0.0853	
Buccal vs. Lingual	*p* = 0.9984		*p* = 0.0082		*p* = 0.2566		*p* = 0.7715		*p* = 0.1802		*p* = 0.8579		*p* = 0.3306		*p* = 0.8871	

* *p* < 0.05 indicates a significant difference between the two groups; SD: Standard Deviation

## Data Availability

The data that support the findings of this study are available from the corresponding author upon reasonable request.

## Supplementary Materials

The following supporting information can be downloaded at: https://www.mdpi.com/article/10.3390/biomedicines11020570/s1, Table S1: Pairwise comparison of the proximal surfaces mean marginal gaps between eight groups by Tukey’s multiple posthoc procedures; Table S2: Pairwise comparison of the axial surfaces with mean marginal gaps between eight groups by Tukey’s multiple posthoc procedures.
