# Supplementary material for: The Effect of a Digital Manufacturing Technique, Preparation Taper, and Finish Line Design on the Marginal Fit of Temporary Molar Crowns: An In-Vitro Study
Source: Biomedicines. 2023 Feb 15;11(2):570. doi: 10.3390/biomedicines11020570 (PMC9953521; doi:10.3390/biomedicines11020570)
Supplement: Supplementary file 1 [file biomedicines-11-00570-s001.zip › biomedicines-2207107-supplementary.pdf]

Table S1: Pair wise comparison of the proximal surfaces mean marginal gaps between eight groups by Tukey's multiple post-hoc procedures.

Group	1		2		3		4		5		6		7		8	
	MD/p-Value		MD/p-Value		MD/p-Value		MD/p-Value		MD/p-Value		MD/p-Value		MD/p-Value		MD/p-Value	
	Mesial	Distal	Mesial	Distal	Mesial	Distal	Mesial	Distal	Mesial	Distal	Mesial	Distal	Mesial	Distal	Mesial	Distal
1	X		−0.13/ 0003*	−0.07/ 0.0060*	0.04\ 0.8240	0.03/ 0.8224	0.01/ 1.0000	0.08/ 0.0016*	−0.00/ 1.0000	0.05/ 0.1173	−0.05/ 0.6995	0.01/ 0.9997	0.04/ 0.8618	0.02/ 0.9420	0.05/ 0.5472	0.04/ 0.3493
2	0.13/ 0003*	0.07/ 0.0060*	X		0.17/ 0.0001*	0.10/ 0.0001*	0.14/ 0.0002*	0.16/ 0.0001*	0.13/ 0.0005*	0.13/ 0.0001*	0.09/ 0.0481*	0.08/ 0.0013*	0.17/ 0.0001*	0.10/ 0.0002*	0.19/ 0.0001*	0.12/ 0.0001 *
3	−0.04/ 0.8240	−0.03 0.8224	−0.17/ 0.0001*	−0.10/ 0.0001*	X		−0.03/ 0.9530	0.05/ 0.1118	−0.04/ 0.7396	0.03/ 0.8920	0.09/ 0.0472*	−0.02/ 0.9748	−0.00/ 1.0000	−0.01/ 1.0000	0.01/ 0.9998	0.01/ 0.9947
4	−0.01/ 1.0000	−0.08/ 0.0016*	−0.14/ 0.0002*	−0.16/ 0.0001*	0.03/ 0.9530	−0.05/ 0.1118	X		−0.01/ 0.9996	−0.03/ 0.8117	−0.06/ 0.4660	−0.07/ 0.0073*	0.03/ 0.9685	−0.06/ 0.0517	0.04/ 0.7735	−0.04/ 0.4642
5	0.00/ 1.0000	−0.05/ 0.1173	−0.13/ 0.0005*	−0.13/ 0.0001*	0.04/ 0.7396	−0.03/ 0.8920	0.01/ 0.9996	0.03/ 0.8117	X		−0.04/ 0.7896	−0.04/ 0.3089	0.04/ 0.7856	−0.03/ 0.7354	0.06/ 0.4474	−0.01/ 0.9993
6	0.05/ 0.6995	−0.01/ 0.9997	−0.09/ 0.0481*	−0.08/ 0.0013*	−0.09/ 0.0472*	0.02/ 0.9748	0.06/ 0.4660	0.07/ 0.0073*	0.04/ 0.7896	0.04/ 0.3089	X		0.08/ 0.0582	0.01/ 0.9974	0.10/ 0.0132*	0.03/ 0.6568
7	−0.04/ 0.8618	−0.02/ 0.9420	−0.17/ 0.0001*	−0.10/ 0.0002*	0.00/ 1.0000	0.01/ 1.0000	−0.03/ 0.9685	0.06/ 0.0517	−0.04/ 0.7856	0.03/ 0.7354	−0.08/ 0.0582	−0.01/ 0.9974	X		0.01/ 0.9995	0.02/ 0.9608
8	−0.05/ 0.5472	0.04/ 0.3493	−0.19/ 0.0001*	0.12/ 0.0001*	−0.01/ 0.9998	−0.01/ 0.9947	−0.04/ 0.7735	0.04/ 0.4642	−0.06/ 0.4474	0.01/ 0.9993	−0.10/ 0.0132*	−0.03/ 0.6568	−0.01/ 0.9995	−0.02/ 0.9608	X	

* $p < 0.05$ indicates a significant difference between the two groups; MD: mean difference (difference in means); X: Not Applicable.

Table S2: Pair wise comparison of the axial surfaces mean marginal gaps between eight groups by Tukey's multiple post-hoc procedures.

Group	1		2		3		4		5		6		7		8	
	MD/p-Value		MD/p-Value		MD/p-Value		MD/p-Value		MD/p-Value		MD/p-Value		MD/p-Value		MD/p-Value	
	Buccal	Lingual	Buccal	Lingual	Buccal	Lingual	Buccal	Lingual	Buccal	Lingual	Buccal	Lingual	Buccal	Lingual	Buccal	Lingual
1	X		−0.00/ 1.0000	−0.12/ 0.0001*	0.04/ 0.1109	0.02/ 0.9660	0.04/ 0.1448	0.03/ 0.8471	0.05/ 0.0204*	0.01/ 0.9999	0.02/ 0.9370	0.00/ 1.0000	0.04/ 0.1181	0.02/ 0.9808	0.04/ 0.1157	0.06/ 0.0809
2	0.00/ 1.0000	0.12/ 0.0001*	X		0.05/ 0.0683	0.14/ 0.0001*	0.05/ 0.0914	0.15/ 0.0001*	0.06/ 0.0112*	0.13/ 0.0001*	0.02/ 0.8663	0.13/ 0.0001*	0.05/ 0.0732	0.14/ 0.0001*	0.05/ 0.0715	0.18/ 0.0001*
3	−0.04/ 0.1109	−0.02/ 0.9660	−0.05/ 0.0683	−0.14/ 0.0001*	X		−0.00/ 1.0000	0.01/ 1.0000	0.01/ 0.9981	−0.01/ 0.9986	−0.03/ 0.7320	−0.02/ 0.9915	−0.00/ 1.0000	−0.00/ 1.0000	−0.00/ 1.0000	0.04/ 0.5622
4	−0.04/ 0.1448	−0.03/ 0.8471	−0.05/ 0.0914	−0.15/ 0.0001*	0.00/ 1.0000	−0.01/ 1.0000	X		0.01/ 0.9945	−0.02/ 0.9747	−0.02/ 0.7998	−0.03/ 0.9317	0.00/ 1.0000	−0.01/ 0.9998	0.00/ 1.0000	0.03/ 0.7956
5	−0.05/ 0.0204*	00.01/ 0.9999	−0.06/ 0.0112*	−0.13/ 0.0001*	−0.01/ 0.9981	0.01/ 0.9986	−0.01/ 0.9945	0.02/ 0.9747	X		−0.04/ 0.3223	−0.00/ 1.0000	−0.01/ 0.9975	0.01/ 0.9996	−0.01/ 0.9977	0.05/ 0.2096
6	−0.02/ 0.9370	−0.00/ 1.0000	−0.02/ 0.8663	−0.13/ 0.0001*	0.03/ 0.7320	0.02/ 0.9915	0.02/ 0.7998	0.03/ 0.9317	0.04/ 0.3223	0.00/ 1.0000	X		0.03/ 0.7484	0.02/ 0.9962	0.03/ 0.7430	0.06/ 0.1365
7	−0.04/ 0.1181	−0.02/ 0.9808	−0.05/ 0.0732	−0.14/ 0.0001*	0.00/ 1.0000	0.00/ 1.0000	−0.00/ 1.0000	0.01/ 0.9998	0.01/ 0.9975	−0.01/ 0.9996	−0.03/ 0.7484	−0.02/ 0.9962	X		0.00/ 1.0000	0.04/ 0.4947
8	−0.04/ 0.1157	−0.06/ 0.0809	−0.05/ 0.0715	−0.18/ 0.0001*	0.00/ 1.0000	−0.04/ 0.5622	−0.00/ 1.0000	−0.03/ 0.7956	0.01/ 0.9977	−0.05/ 0.2096	−0.03/ 0.7430	−0.06/ 0.1365	−0.00/ 1.0000	−0.04/ 0.4947	X	

* $p < 0.05$ indicates a significant difference between the two groups; MD: mean difference (difference in means); X: Not Applicable.
